# Reducing privacy risks of China’s healthcare big data through the policy framework

**DOI:** 10.3389/fpubh.2024.1414076

**Published:** 2024-07-03

**Authors:** Xinyuan Shi

**Affiliations:** School of Law, Xiamen University, Xiamen, China

**Keywords:** healthcare big data, privacy risks, policy framework, privacy impact assessment, Chinese experience

## Abstract

While healthcare big data brings great opportunities and convenience to the healthcare industry, it also inevitably raises the issue of privacy leakage. Nowadays, the whole world is facing the security threat of healthcare big data, for which a sound policy framework can help reduce privacy risks of healthcare big data. In recent years, the Chinese government and industry self-regulatory organizations have issued a series of policy documents to reduce privacy risks of healthcare big data. However, China’s policy framework suffers from the drawbacks of the mismatched operational model, the inappropriate operational method, and the poorly actionable operational content. Based on the experiences of the European Union, Australia, the United States, and other extra-territorial regions, strategies are proposed for China to amend the operational model of the policy framework, improve the operational method of the policy framework, and enhance the operability of the operational content of the policy framework. This study enriches the research on China’s policy framework to reduce privacy risks of healthcare big data and provides some inspiration for other countries.

## 1 Introduction

Data has become a ubiquitous concept in our daily lives with massive amounts of data being collected, stored, processed, and analyzed on a daily basis. This characterization is cross-disciplinary, ranging from the fields of machine learning and engineering to economics and healthcare (1). In the healthcare industry, the explosion of data provides a tremendous opportunity to gather insights that can improve patient outcomes and even save lives (2). This kind of data can be referred to as “healthcare big data.” According to Article 4 of the Notice on National Healthcare Big Data Standards, Security and Service Management Measures in China (for Trial Implementation) issued by the National Health Commission on July 12, 2018, healthcare big data refers to healthcare-related data generated in the course of people’s disease prevention, treatment, and health management. Healthcare big data has enormous potential to improve patient outcomes, predict epidemic outbreaks, gain valuable insights, avoid preventable diseases, reduce the cost of healthcare delivery, and improve overall quality of life (3). The Guiding Opinions on Promoting and Regulating the Application and Development of Healthcare Big Data, issued by the General Office of the State Council of China on June 21, 2016, pointed out that healthcare big data is an important basic strategic resource of the country. By the end of 2023, more than 80% of tertiary hospitals in 30 provinces in China have realized the acceptance and application of e-health cards (codes), with a population coverage rate of nearly 70%. Currently, 100 percent of provinces in China have established regional universal health information platforms (4).

According to China’s Information Security Technologies - A Guide to Healthcare Data Security (GB/T 39725–2020), healthcare big data consists of six categories, which are personal attribute data, health status data, medical application data, healthcare payment data, health resource data, and public health data. First, personal attribute data is data that can identify a specific natural person, either alone or in combination with other information. Second, health status data is data that reflect or are closely related to the health of an individual. Third, healthcare application data is data that reflects healthcare, outpatient visits, hospitalizations, discharges, and other healthcare services. Fourth, healthcare payment data refers to cost-related data involved in services such as healthcare or insurance. Fifth, health resource data is data that can reflect the capacity and characteristics of health providers, health programs, and health systems. Sixth, public health data is data related to the health of the general public in a country or region.

Big data analysis in healthcare offers many benefits, prospects, and great potential for healthcare transformation, but it also presents multiple obstacles and challenges. Privacy protection is one of the most important and challenging tasks in healthcare (5). The ability “to protect individual privacy in the era of big data has become limited” (6). As mentioned above, healthcare is awash in valuable data. Every patient, test, scan, diagnosis, treatment plan, medical trial, prescription, and ultimate health outcome produces a data point that can help improve how care is given in the future. Typically, a large amount of data is called “big data” and it’s through these vast amounts of data that some of the biggest possible health advances lie. Big data refers to large data sets consisting of both structured and unstructured data that are analyzed to find insights, trends, and patterns. Most commonly, big data is defined by the three V’s-volume, velocity, and variety-meaning that it has a high volume of data that is generated quickly and consisting of different data types, such as text, images, graphs, or videos (7). The above characteristics of big data have led to the failure of traditional healthcare data’s efforts toward anonymization. This is because the combination of different types of data encompassed by big data in healthcare can make the process of identifying an individual very easy. This risk is already beginning to manifest, as can be seen in research involving genetic samples and genetic data. Researchers have shown that for the vast majority of Americans, de-identified genetic data can be reattached to the identity of the person who provided the initial samples. This reattachment of identity to data is accomplished via family maps created by public genealogy databases (8). Apparently, the application of big data in healthcare poses even more serious privacy risks. In the U.S., an analysis of data breaches recorded in privacy databases between 2015 and 2022 shows that 32% of all recorded data breaches occurred in the healthcare sector, which is almost double the number of breaches recorded in the financial and manufacturing sectors. This is because healthcare big data is more valuable on the black market than any other type of data. For example, it takes longer to detect healthcare fraud than stolen credit cards, and breaches can be stopped as soon as they are detected (9). Therefore, in the field of healthcare, how to prevent data leakage and protect personal privacy has become an important frontier research topic that needs to be solved in the process of informatization construction and development today.

A sound policy framework can help reduce privacy risks of healthcare big data. “Policy is a process of purposeful activity, undertaken by one or a group of actors to deal with an issue or a related matter” (10). According to this definition, the manifestations of policy include laws and regulations, industry standards, self-regulatory rules, and other forms. Since 2013, China has introduced several policies to promote the development of the healthcare big data industry, and these policies include the rules to reduce privacy risks. Although the issue of reducing privacy risks of China’s healthcare big data through policies has received extensive attention both at home and abroad, on the whole, most of the relevant studies have been limited to the policy operational model, while neglecting to consider and reflect on the policy operational method and the policy operational content. For example, Su points out that China should build a multifaceted policy framework that combines national legislation with other social norms to reduce privacy risks of healthcare big data (11). Qin et al. (12) argued that China is still relatively backward in the formal legislative protection of patient information privacy compared with developed countries, and further suggested that the formal legislative work of patient privacy protection should be vigorously promoted. Given this, this paper systematically constructs a Chinese policy framework for reducing privacy risks of healthcare big data, which includes the policy operational model, the policy operational method, and the policy operational content, analyzes the limitations of the policy framework and puts forward corresponding countermeasure suggestions on this basis, so as to make up for the shortcomings of the existing research.

The remainder of this paper is organized as follows. Privacy issues raised by healthcare big data belong to the coming section, followed by the discussion of China’s policy framework for reducing privacy risks of healthcare big data and its shortcomings. The recommendation section is the last, where we propose specific countermeasures to improve China’s policy framework for reducing privacy risks of healthcare big data. On the one hand, from the perspective of the research purpose, this study aims to reflect on the shortcomings of China’s policy framework for reducing privacy risks of healthcare big data, specifically pointing to the problems of the policy operational model, the policy operational method and the policy operational content, and then proposing the improvement of the policy framework for reducing privacy risks of healthcare big data. On the other hand, from the perspective of research value, some countries similar to China, especially developing countries, are also experiencing the dilemma of the policy framework for reducing privacy risks of healthcare big data, and this study can provide better insights and suggestions for these countries to construct a reasonable policy framework for reducing privacy risks of healthcare big data.

## 2 Privacy concerns in healthcare big data

Chinese legislation positions healthcare big data as sensitive personal information. According to paragraph 2 of Article 28 of the Personal Information Protection Act (PIPA), sensitive personal information refers to the personal information that, if disclosed or illegally used, could easily lead to infringement of a natural person’s human dignity or jeopardize the safety of his or her body or property, including information on biometrics, religious beliefs, specific identities, medical care and health care, financial accounts, whereabouts and trajectories, and personal information on minors who are less than 14 years of age. Therefore, privacy protection of healthcare big data is essentially the protection of sensitive personal information.

With the rapid growth of healthcare big data, its increasing exposure to privacy risks is a concern (13). Today, the entire world is facing security threats to healthcare big data.

In the U.S., the number of healthcare big data breaches has been increasing essentially every year. In 2023, more than 540 organizations reported healthcare big data breaches to the U.S. Department of Health and Human Services (HHS)’ Office for Civil Rights, impacting upwards of 112 M individuals, which means that one-third of Americans have privacy issues with their healthcare big data (14). The HCA Healthcare Big Data Breach, which resulted in the loss of privacy for 11,270,000 patients, is a typical example. HCA Healthcare is a large healthcare organization comprised of 180 hospitals and 2,300 ambulatory sites of care in 20 states and the United Kingdom. The breach occurred when an unauthorized party stole a list of information used for email messages to patients and posted it on an online forum. The list contained information used for email messages, such as appointment reminders and education about healthcare programs and services. The list consisted of 27 million rows of data. HCA Healthcare stated that the incident “appears to be a theft from an external storage location exclusively used to automate the formatting of email messages,” and it caused no disruptions to operations or care. The list contained patient names, cities, states, zip codes, email addresses, phone numbers, gender, dates of birth, and appointment information (14).

In the UK, the series of The National Health Service (NHS) data breaches between July 2011 and July 2012 was one of the largest incidents affecting the UK healthcare industry. The NHS is a publicly-funded healthcare system in England. The security breaches took place across multiple units of the National Health Service, and NHS Surrey is one of those multiple parts. NHS Surrey was fined by The Information Commissioner’s Office (ICO) £200,000 when it was found that over 3,000 patient records had been discovered online. The security breach was the result of secondhand NHS computers that had been auctioned off on eBay, ones that the data and hardware destruction company had failed to destroy properly. The ICO also found three additional NHS computers containing sensitive patient information, all of which had been sold online. The responsibility was still under NHS Surrey for failing to monitor and check with their third-party service provider that records had been properly destroyed (15).

In China, healthcare big data breaches are increasing every year, and as many as 902,529,000 data leaks in the healthcare industry occurred in 2023. The Jiaozhou Hospital data breach that happened in 2020 is even more widely debated. On April 16, 2020, internal staff at the Jiaozhou Central Hospital in Qingdao widely disseminated the names, telephone numbers, identity card numbers, personal details of residential addresses and types of consultations of 6,685 patients to WeChat groups, which led to serious disruptions in the personal lives of the patients on the list, and even rumors that some of the patients were infected with COVID-19 as a result (16).

It was found that the main disclosure types of protected healthcare information were hacking incidents, unauthorized access (internal), theft or loss, and improper disposal of unnecessary data (17). The different disclosure types mentioned above are briefly explained below:

Hacking incidents: hacking incidents comprise all cyber-attacks that are used to gain unauthorized access to confidential data. Ransomware and malware are the main approaches that are used to expose protected health information.

Unauthorized access (internal): these includes all types of attacks that lead to the exposure of confidential health data with the help of any internal source of an organization. This may be abuse of privileges, unauthenticated access/disclosure, etc.

Theft or loss: this comprises all incidents that lead to the disclosure of protected health information in the form theft or loss, such as the theft of hard disks, laptops, or any other portable device that contains protected healthcare data. This can also be because of catastrophic damage or the loss of these devices.

Improper disposal of unnecessary data: unnecessary but sensitive and confidential data should be properly disposed of so that it cannot later be retrieved. Improper disposal of this data can lead to the disclosure of protected health information. Improper disposal attack type includes all breached incidents that are caused by the improper disposal of unnecessary but sensitive and confidential health data.

However, in the last three or 4 years, theft/loss and improper disposal have shown a decreasing trend. In contrast, hacking/IT incidents and unauthorized internal disclosures have shown a marked increases, especially hacking incidents, which have increased very rapidly in frequency in last few years (17).

As a result, the following hazards will arise with the leakage of healthcare big data and the violation of sensitive personal information:

First, it endangers patients’ health. There is a possibility that the physical health data recorded by patients through wearable devices may be deleted and modified, and when these already tampered data are applied to patients’ treatment, it will be harmful to their health. In addition, the leakage of healthcare big data may aggravate the psychological burden and fear of patients, thus affecting the process and effect of their treatment (18). Some healthcare big data leakage may affect patients’ lives, and may even lead to patients’ suicide.

Second, it affects the safety of patients’ property. After the information of some seriously ill patients has been illegally obtained, patients may be induced to buy the so-called “miracle drugs” under the psychological effect of the patient’s urgency to seek medical treatment, which affects the normal treatment of the patient and also aggravates the patient’s financial burden (19).

Third, it violates patients’ human dignity. Healthcare big data contains patients’ basic information, past medical history, medication records, health information, etc. Once leaked, such information may cause discrimination against patients and infringe upon their human dignity, thus affecting their work and life. For example, if the condition of an AIDS patient is disclosed, the patient may be discriminated against by others, which may aggravate the patient’s condition.

## 3 Discussion: China’s policy framework for reducing privacy risks of healthcare big data and its problems

So far, the development of China’s healthcare information technology has experienced the following three stages: the first stage began in the 1980s. At this stage, the construction of informatization was dominated by a single large public hospital, and the hospital information system was mostly used for cost settlement; the second stage began in 2003 during the “SARS” prevention and treatment period. At this stage, the state increased its investment in public health informatization, and a large number of government-led health emergency command systems and direct reporting systems for health statistics were built and applied successively; the third stage is marked by the national “new healthcare reform” in 2009. At that stage, informatisation was written into the national healthcare reform report as an important support system (20).

Since 2016, the Chinese legislature and relevant institutions have issued a series of policy documents on reducing privacy risks of healthcare big data (Table 1), which have improved China’s legal safeguard capacity for the protection of privacy of healthcare big data. However, there are still some problems and shortcomings with the above policy framework for reducing privacy risks of healthcare big data.

**Table 1:** A series of policy documents on reducing privacy risks of healthcare big data in China.

Issuing date	Issuing institutions	The policy documents
June 2016	General Office of the State Council	Guiding Opinions on Promoting and Standardizing the Development of Healthcare Big Data Applications
October 2016	Central Committee of the Communist Party of China; General Office of the State Council	Outline of the “Healthy China 2030” Plan
January 2018	State Food and Drug Administration	Guiding Principles for Technical Review of Mobile Medical Device Registrations
April 2018	General Office of the State Council	Opinions on Promoting the Development of “Internet+ Healthcare”
July 2018	The National Health Commission	National Healthcare Big Data Standards, Security and Service Management Approach (Trial)
August 2018	The National Health Commission	Circular on Further Promoting the Informatization of Medical Institutions with Electronic Medical Records at its Core
December 2019	Standing Committee of the Thirteenth National People’s Congress	Basic Medical Care and Health Promotion Act
October 2020	The General Administration of Market Supervision and Administration; the Standardization Administration	Information Security Technology -- Specification for the Security of Personal Information(GB/T 35273–2020)
December 2020	The General Administration of Market Supervision and Administration; the Standardization Administration	Information Security Technologies - A Guide to Healthcare Data Security(GB/T 39725–2020)
April 2021	National Healthcare Security Administration	Guiding Opinions on Strengthening Network Security and Data Protection Work
July 2021	Internet Society of China、Related Enterprises in the Internet Medical and Healthcare Industry Alliance、Healthcare Institutions	Self-Regulation Convention on Healthcare Network Data Security
August 2021	Standing Committee of the Thirteenth National People’s Congress	Personal Information Protection Act
September 2021	Standing Committee of the Thirteenth National People’s Congress	Data Security Act
March 2022	Central Committee of the Communist Party of China; General Office of the State Council	Opinions on Strengthening Ethical Governance of Science and Technology
August 2022	The National Health Commission	Measures for the Management of Network Security in Healthcare Institutions
February 2023	the National Health Commission; Ministry of Education; Ministry of Science and Technology; Administration of Traditional Chinese Medicine	Measures for Ethical Review of Life Sciences and Medical Research Involving Human Beings

### 3.1 The operational model of the policy framework is mismatched

China’s policy framework for reducing privacy risks of healthcare big data adopts a general data protection operational model, which makes the relevant policies insufficiently targeted and specialized to address practical issues. To summarize, global policy frameworks for reducing privacy risks of healthcare big data present the following three main operational models (21): The first is the operational model of general data protection, a type of legislation that is usually based on constitutional human rights principles for the comprehensive protection of individual privacy. Typical examples are the European Union’s General Data Protection Regulation, the Canadian Personal Information Protection and Electronic Documents Act, and the German Data Protection Act. The second is a specialized operational model for privacy protection in the healthcare sector. Typical examples are the U.S. Health Insurance Portability and Accountability Act of 1996, the U.S. Health Information Technology for Economic Clinical Health Act, the Australian Health Records and Information Privacy Act 2002 of New South Wales, the French Medical Privacy Act, and the French Healthcare Insurance Act. The third is the operational model of industry self-regulation. Some countries, in order to encourage and facilitate the development of the network industry, tend to rely on self-regulation from network service providers and social monitoring from industry associations to achieve privacy governance. For example, the U.S. Department of Commerce developed the Effective Self-Regulation for Protection of Privacy as early as 1998. In addition, there are a number of industry consortia in the United States that have issued privacy guidelines, such as the Online Privacy Alliance, the Technology Protection Model, the Personal Privacy Authentication, and the Patient Privacy Rights Group (22).

China’s current policy framework mainly adopts a general data protection operating model, lacking specialized legislation and industry self-regulatory rules to reduce privacy risks of healthcare big data. Specifically, although the Guiding Opinions on Promoting and Standardizing the Development of Big Data Applications in Healthcare, National Healthcare Big Data Standards, Security and Service Management Measures, etc. have been issued, the above documents are departmental administrative regulations rather than authoritative laws, which makes their authority and coercive power very limited. In addition, the Internet Society of China, in conjunction with the Internet Medical and Healthcare Industry Alliance signed the Self-Regulation Convention on Healthcare Network Data Security, but the overall policy provisions are too general and rough, and there are no clear and detailed provisions on self-regulatory standards for healthcare network data security, data security assessment and verification, functions of public service platforms and other related supporting measures.

### 3.2 The operational method of the policy framework is inappropriate

Most of China’s policies for reducing privacy risks of healthcare big data focus on the assumption of legal liability after the fact, while neglecting the ex-ante prevention mechanism. As a matter of fact, once the healthcare big data is leaked, it will cause great damage to the patients’ personality and property. For this reason, it is crucial to establish the ex-ante prevention mechanism to mitigate privacy risks. Overall, the ex-ante preventive mechanism consists of two main elements.

#### 3.2.1 The privacy impact assessment (PIA) mechanism

In the field of healthcare, according to the HHS, the PIA refers to the need for businesses and organizations to assess the potential negative impact of the collection, use, sharing, and maintenance of personal health information on the privacy of individuals, as well as the need for the HHS to publish the PIA report and the relevant privacy protection policies of the enterprises and organizations on a platform accessible to the public after the assessment is completed (23). It should be clear that the PIA is more than just a compliance check, it is also designed to identify and minimize privacy risks (24). Many healthcare big data applications are designed with privacy concerns, such as the U.S. Cancer Moonshot program. This program uses large amounts of data on treatment plans and recovery rates of cancer patients in order to find trends and treatments that have the highest rates of success in the real world. For example, researchers can examine tumor samples in biobanks that are linked up with patient treatment records. Using this data, researchers can see things like how certain mutations and cancer proteins interact with different treatments and find trends that will lead to better outcomes (25). As a matter of fact, if the data of cancer patients are leaked, it will cause their psychological burden and aggravate their conditions, so it is necessary to build a privacy impact assessment mechanism to prevent the problem before it occurs. However, on the one hand, the Chinese government has not yet established an open sharing platform for privacy policies, and on the other hand, in terms of legal policies, there are only rough provisions on the PIA in the Article 55 and 56 of PIPA, and there is a lack of standards and rules specifically for the PIA of healthcare big data.

#### 3.2.2 The ethics review mechanism

The ever-developing network technology has brought the information ethics issues such as network security and personal privacy, which pose a great challenge to the subjective status and autonomy of human beings. Given this, the formation of a specialized ethics review body and its review of the impact of healthcare big data applications on personal privacy can better control the risk of personal privacy infringement at the source (26). In this regard, there have been similar experiences overseas. For example, in Australia, the National Statement on Ethical Conduct in Human Research 2023 stipulates that the collection, storage and analysis of personal health data in the course of research requires the approval of the ethics review body. It is worth noting that the Article 5 of the Measures for Ethical Review of Life Sciences and Medical Research Involving Human Beings issued by China’s National Healthcare Commission, Ministry of Education, Ministry of Science and Technology and Administration of Traditional Chinese Medicine in February 2023 clearly stipulate that ethics review committees should be set up in relevant medical and health institutions, as well as in schools of higher education and scientific research institutes, where life sciences and medical research involving human beings is conducted. The committee is responsible for conducting ethical review of life sciences and medical research involving human beings, and regularly provides bioethics education and training to researchers, students, research administrators and other relevant personnel engaged in life sciences and medical research involving human beings. Regrettably, this policy document is still relatively simple and rough, and the provisions on the organizational structure mechanism, talent training mechanism, operational safeguard mechanism and file management mechanism of the Ethics Review Committee need to be refined and perfected.

### 3.3 The operational content of the policy framework is poorly actionable

The application of healthcare big data carries privacy risks, and the ethical principle for its specific challenges is respect for the autonomy of the participants (27). The primary method of respecting individual autonomy is to obtain informed consent, ensuring that patients understand the purpose, risks, and methods of the project being undertaken. The consent process upholds ethical principles of autonomy and freedom of choice by allowing patients to make a well-informed decision. The“informed-consent” rule has been the best tool for upholding the ethical principle of respect for participant autonomy (28). In this regard, although China’s “informed-consent”rule is formally characterized as respecting participant autonomy, it fails to truly protect the privacy of participants. Specifically, In China, PIPA is the core policy for big data privacy protection in healthcare, with “informed-consent” as the core processing rule. Specifically, in accordance with articles 13 and 14 of PIPA, a processor of personal information must obtain the consent of the provider of the personal information before processing the information. Furthermore, such consent shall be given voluntarily and explicitly by the personal information provider with full knowledge. In the healthcare field, the “informed-consent” rule means that hospitals, as well as disease prevention and control organizations should inform the data providers of what information they need and how it will be processed and used before collecting it, and the personal healthcare big data can only be processed and used with the explicit consent of the data provider. In essence, however, the “informed-consent” rule is set up in such a way that it affects the flow of data and does not truly protect the privacy of individuals. On the one hand, for data users, if every act of information collection or use requires the consent of the data provider, it will seriously increase the legitimate cost of data use, which will in turn affect the flow of data and treatment outcomes (29). For example, Optum Labs, a US research collaborative, has collected Electronic Health Records (EHRs) of over 30 million patients to create a database for predictive analysis tools that will improve the delivery of care. This would help doctors make data-driven decisions within seconds and improve patients’ treatment, which is particularly useful in the case of patients with complex medical histories suffering from multiple conditions (28). However, obtaining prior consent from the data provider for each prediction activity would significantly reduce the effectiveness of disease treatment. On the other hand, the logic of the “informed-consent” rule is that data providers can rationally self-manage their personal information, so as to safeguard their interests. However, empirical studies have shown that even if the text of a privacy policy is easy to understand, users rarely read privacy policies carefully (30), resulting in a much lower likelihood of being “informed.”In conclusion, the legislator’s original intent in establishing the “informed-consent” rule is far from being fulfilled.

## 4 Suggestions

### 4.1 Adjusting the operational model of the policy framework

At present, China’s policy framework for the privacy protection of healthcare big data mainly adopts the general data protection operational model, resulting in a lack of specialization and relevance. In this regard, it is recommended that China, on the basis of the existing operational model, explore and develop the specialized operational model and the industry self-regulation operational model, so as to strengthen the systemic and professional nature of China’s policy framework for big data privacy protection in healthcare. Specifically:

On the one hand, the enactment of a specialized law is imperative in the long run. Systematically, China’s existing policy framework for healthcare big data privacy governance is still in a decentralized state, and does not reflect systematic, targeted and authoritative, so it is necessary to learn from the experience of the United States, Australia and France to formulate a special law to regulate and reduce the risk of healthcare big data privacy, for example, to formulate a Healthcare Privacy Law. Of course, the enactment of this special law is not a quick fix; it is a systematic project involving political, legal, cultural, technological and market factors, which needs to be explored and optimized in the light of practical experience. Specifically, first, China’s healthcare informatization started relatively late compared to developed countries such as the United States, Australia, and France, which means that regulatory strategies and technical standards related to reducing the privacy risks of healthcare big data have yet to be explored and optimized based on the accumulation of practical experience. While according to China’s legislative experience, the introduction of laws often requires a cumbersome and time-consuming process. In view of this, the bottom-up legislative path is more in line with China’s national conditions. Additionally, China, as the world’s second country in terms of population, has distinctly different development conditions, cultural backgrounds, and interests among its provinces and ethnic groups, which poses a serious challenge to the formulation of a specialized law to reduce the privacy risks of healthcare big data. Therefore, the model of local pilot legislation is more in line with China’s national conditions. Taking all these factors into account, this paper concludes that the following principles should be followed in the formulation of the special legislation on healthcare big data privacy governance:

First, the principles should precede and then be elevated to law. Because of the complexity and technicality of the management of healthcare big data in the era of big data, the corresponding guiding principles should be issued first, and then be elevated to legal rules when the time is ripe. For example, China’s Food and Drug Administration (FDA) issued the first Guiding Principles for Technical Review of Registration of Mobile Medical Devices in 2018, which together with the Guiding Principles for Technical Review of Registration of Medical Device Software that have been previously issued, regulate the collection and use of healthcare big data in the field of mobile medical devices in China.

Second, the standards should precede and then be elevated to law. Healthcare big data needs to be regulated by numerous technical standards on its collection and use behavior. For example, the “Information Security Technology - Personal Information Security Standards” (GB/T 35273–2020) issued by the China General Administration of Market Supervision and Administration and the Standardization Administration of China in 2020 defines for the first time the concepts of personal information and express consent, and clarifies the specific requirements for the collection, preservation, use, entrusted processing, sharing, transfer, and public disclosure of personal information, and also provides templates for “examples of personal information, ““determination of sensitive personal information,” “methods for safeguarding the right of the consent of the subject of personal information,” and “privacy policy.” Since the introduction of the “Information Security Technology - Personal Information Security Standards” (GB/T 35273–2020), Chinese apps such as WeChat, Taobao, Alipay, and Sina Weibo have updated their privacy policies in accordance with it (31). It can be seen that this policy document is a national recommendatory standard without national mandatory power, but it essentially guides and constrains the behavior of enterprises in the use of information.

Third, local legislation should precede and then be elevated to national law. Healthcare big data involves the data management and privacy protection demands of different groups in different regions, so the time is not yet ripe for the introduction of national general law. This paper argues that national legislation should be formulated on the basis of positive exploration and pilot testing of local legislation. As a matter of fact, China has been exploring local legislation on healthcare big data in recent years along this line of thought and path. For example, in February 2017, the government of Guangdong Province issued the Implementing Opinions on Promoting and Regulating the Development of Healthcare Big Data Applications; in April 2017, the Fuzhou Government issued the Interim Measures for the Management of Healthcare Big Data Resources in Fuzhou; In September 2018, the Standing Committee of the Guiyang People’s Congress issued the Regulations on the Development of Big Data Application for Healthcare in Guiyang; In August 2020, the government of Shandong Province issued the Measures for the Management of Healthcare Big Data in Shandong Province, etc. These local legislations mentioned above provide important legal safeguards for the interconnection and sharing of local healthcare big data and privacy protection, as well as important references and experiences for future national legislations.

On the other hand, the system of industry self-regulatory rules for healthcare big data privacy protection in China should be enriched and expanded. As mentioned earlier, the only self-regulatory rule on healthcare big data privacy protection in China is the Self-Regulatory Convention on Healthcare Network Data Security, but the rules within it are too rough and simple. It is recommended that more self-regulatory rules be issued in the future to cover all aspects of healthcare big data privacy protection as far as possible, such as the Self-Regulatory Convention on Privacy Compliance Management of Healthcare Big Data, the Self-Regulatory Convention on the Construction of Healthcare Big Data Platforms, and the Self-Regulatory Convention on the Risk Assessment of Privacy of Healthcare Big Data, etc., so as to give full play to the dynamic role of industry self-regulation supervision.

### 4.2 The operational method of the policy framework should be improved

In order to minimize privacy risks of healthcare big data, it is necessary to establish a pre-emptive mechanism.

**Figure 1:**
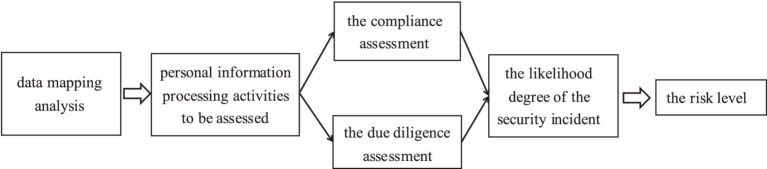
Privacy impact assessment process for big data in healthcare.

On the one hand, the State should introduce a special policy on the PIA of healthcare big data. The PIA of healthcare big data can assess and identify the “high-risk” parts of the whole life cycle of healthcare big data, which can help hospitals or third-party organizations adjust or transfer the “high-risk” processing activities, thereby reducing or avoiding the risk of non-compliance due to the aforementioned erroneous processing and protecting patients’ privacy. Specifically, a policy on the PIA of healthcare big data should focus on building the PIA process that includes the following steps (Figure 1) (32):

- Data Mapping. Identify the corresponding healthcare big data processing activities in conjunction with the full data lifecycle and interview instruments.
- Conduct the PIA of healthcare big data processing activities. The assessment broadly consists of two parts: a compliance assessment and a due diligence assessment (33). Compliance assessment refers to the assessment of whether the purpose of the processing of healthcare big data and the manner of processing are lawful, legitimate and necessary, as well as their possible impact on the rights and interests of individuals. The due diligence assessment refers to the assessment of whether the protection measures adopted are lawful, effective and commensurate with the level of risk.
- Evaluate the likelihood of the security incident occurring in the healthcare big data processing activity. The above steps are combined to identify the sources of risk involved in the healthcare big data processing activity and to analyze the likelihood of the security incident occurring, so as to deduce the risk level of the healthcare big data processing activity.
- Publishing the report. At this stage, it is necessary to rely on the open sharing platform for the healthcare big data privacy policy set up by the government, so that users can query the relevant healthcare big data privacy impact assessment report at any time, thus enhancing users’ control over their health information.

On the other hand, the ethical review mechanism for healthcare big data privacy protection should be improved. As mentioned earlier, the Measures for Ethical Review of Life Sciences and Medical Research Involving Human Beings has initially established a basic policy framework for ethical review, but the provisions within it are too rough. It is recommended that specific rules be issued to refine this policy, in particular, more in-depth provisions on the organizational structure mechanism, the talent training mechanism, the operation guarantee mechanism and the file management mechanism of the Ethics Review Committee (34). As a matter of fact, before the introduction of the Measures for Ethical Review of Life Sciences and Medical Research Involving Human Beings, medical and healthcare practice in China had been developing an ethical review mechanism for many years, and a lot of experience had been accumulated. For example, the Ethics Review Committee of Southwest Hospital in China was established as early as 1997, and has been renewed a total of six times, maintaining a sustainable and healthy development trend. The Ethics Review Committee of Southwest Hospital obtained the WHO/SIDCER international certification in May 2010, becoming the first ethics review committee in western China to obtain such certification (35). After years of development, the committee has become more mature in the construction of organizational structure mechanism, talent training mechanism, operation guarantee mechanism and file management mechanism, and it is recommended that the relevant legislative departments in China combine practical experience to refine and improve the ethics review mechanism for the privacy protection of healthcare big data.

### 4.3 The operational content of the policy framework should be strengthened

While strict healthcare big data privacy regulations are more beneficial to the protection of individual privacy, stringent “informed-consent” rules can also affect the flow of data. Since the 18th CPC National Congress, the CPC Central Committee has attached great importance to the development of digitalization from the perspective of the overall situation of national development, and the promotion of high-quality development of the digital economy has been elevated to become a national strategy. Therefore, promoting the flow of data has become an important national task. Of course, this does not mean that individual rights and interests centered on personal privacy should give way to collective rights and interests; the key is how to effectively balance collective and individual interests through the policies. Given this, it is recommended that the privacy protection of healthcare big data implement the “scenario concept,” i.e., to transform the traditional framework of “informed consent” and recognize that the judgment standard of the reasonable use of big data in healthcare depends on whether it meets the reasonable privacy expectation of users and whether it creates unreasonable privacy risks, rather than rigidly examining whether the consent of the person concerned has been obtained. In fact, the implementation of this “scenario concept” still relies on the PIA mechanism for healthcare big data mentioned above. Specifically, when the assessment result is that the impact on an individual’s privacy is minimal, the healthcare big data of an individual can be processed without his/her consent, thus weakening the over-reliance on user consent for the legitimacy of the use of healthcare big data, and balancing the relationship between the flow of data and the privacy protection. In addition, the regulation of healthcare big data application also includes the purpose specification and the security safeguards (36), which are likewise in need of continuous improvement in China’s policies. For example, the purpose specification requires the purposes for which personal data are collected should be specified not later than at the time of data collection and the subsequent use limited to the fulfillment of those purposes, and the security safeguards requires personal data should be protected by reasonable security safeguards against such risks as loss or unauthorized access, destruction, use, modification, or data disclosure.

## 5 Conclusion

Big data offers unlimited opportunities to advance health research, knowledge discovery, clinical care and personal health management. However, big data privacy is recognized as a huge obstacle for researchers in this field (37). This study analyzes several ways that may trigger big data privacy risks in healthcare and their harmful consequences, compiles and discusses the limitations of China’s policy framework for reducing privacy risks of healthcare big data, such as the mismatched operational model, the inappropriate operational method, and the poor actionable operational content. The strategies to refine and optimize the operational model, the operational method, and the operational content are proposed. Similar to China, many other countries also face the dilemma of the shortcomings of the policy framework for reducing privacy risks of healthcare big data. Therefore, this study provides good enlightenment for other countries to minimize privacy risks of healthcare big data and protect patient privacy.

The strategies and technological approaches proposed in this paper may be far from enough to change the status quo of privacy leakage of healthcare big data, but they are significant for enhancing the awareness of the social responsibility of healthcare organizations and safeguarding the public’s right to privacy. Given that reducing privacy risks of healthcare big data through policy frameworks is a constant battle, the government and industry sectors have a long way to go to make and revise policies continuously for reducing privacy risks of healthcare big data.

## Data availability statement

The datasets presented in this article are not readily available because no dataset available. Requests to access the datasets should be directed to sxinyuan04@hotmail.com.

## Author contributions

XS: Writing – original draft, Writing – review & editing.
